# Safe sex negotiation and HIV risk reduction among women: A cross-sectional analysis of Burkina Faso 2021 Demographic and Health Survey

**DOI:** 10.1371/journal.pgph.0003134

**Published:** 2024-04-24

**Authors:** Sulemana Ansumah Saaka, Cornelius K. A. Pienaah, Zakara Stampp, Roger Antabe

**Affiliations:** 1 Department of Geography and Environment, University of Western Ontario, London, Canada; 2 Department of Health and Society, University of Toronto Scarborough, Toronto, Canada; University of Ghana, GHANA

## Abstract

Women are biologically more susceptible to the Human Immunodeficiency Virus (HIV) and other sexually transmitted Infections (STIs) because receptive sex is riskier than insertive. Despite condom use being the staple preventive method for HIV infection (over 80% efficacy), in Sub-Saharan African countries like Burkina Faso, a high burden of HIV and the unmet need for condom use coexist. Moreover, even though women in SSA are disproportionately HIV positive, they are reportedly less capable of negotiating condom use for HIV risk reduction. Thus, using the Health Believe Model (HBM), this study explored the factors that influence condom use among women within the context of HIV prevention, with a key interest in condom use negotiation. Using the women’s dataset of the 2021 Burkina Faso Demographic and Health Survey and applying logistic regression models, this study examined the factors associated with condom use for HIV risk reduction. Women who had confidence to negotiate condom use with their partners (OR = 1.57, P<0.001, 95%CI: 1.29, 1.91), those with secondary education (OR = 1.38, P<0.05, 95%CI: 1.07 1.77), from richest households (OR = 1.64, P<0.05, 95%CI: 1.08, 2.47), the employed (OR = 1.23, P<0.05, 95%CI: 1.02, 1.49), women with knowledge of sexually transmitted infections (OR = 1.58, P<0.001, 95%CI: 1.26, 1.97), those who have ever been tested for HIV (OR = 1.85, P<0.001, 95%CI: 1.52 2.24), as well as those who knew that a healthy-looking person can have HIV (OR = 2.64, P<0.001, 95%CI: 2.15, 3.24) were all significantly more likely to practice condom use for HIV risk reduction. Also, religion and geographical location of participants significantly predicted condom use for HIV risk reduction in the study context. The ability to negotiate condom use, knowledge of HIV and STIs, the socioeconomic status of women, as well as their geographical location, influence their practice of safer sex for HIV risk reduction in Burkina Faso.

## 1.0 Introduction

In Sub-Saharan Africa (SSA), women and girls account for a disproportionate burden of all new HIV infections [1]. Women’s increased biological susceptibility to the virus through sexual intercourse has led to the use of the term “feminization” of the HIV pandemic by United Nations agencies, a term that is more obvious in Sub-Saharan Africa (SSA) than elsewhere in the world. Preventive approaches are crucial for the reduction of HIV transmission and spread of other sexually transmitted infections (STIs). For several decades now, condom use has been the primary recommended method for protection against HIV and STIs. Even though international attention has recently been drawn towards emerging biomedical prevention strategies, such as pre-exposure prophylaxis (PrEP), condom use remains one of the most effective and widely used approaches for preventing sexual transmission of HIV [2]. For instance, evidence from available studies suggests that the efficacy of PrEP for HIV prevention is between 44% and 75% [3, 4]. On the other hand, consistent condom use is approximately between 80%-95% effective for protection against HIV infection [5–8]. Although the promotion of correct and consistent condom use is key to the prevention of sexual transmission of HIV, access to condoms, lack of dialogue among partners regarding condom use/partner objection, inadequate sex education, discomfort/displeasure of condom use, perceived ineffectiveness of condoms, religious and moral values, and gender inequalities are commonly highlighted as barriers to condom use [9].

Particularly in SSA countries like Burkina Faso, gendered disparities are frequently reported with regard to condom use [10–13]. These differences have associations with factors such as socioeconomic status [14], infection risk perceptions [15], HIV knowledge [16], knowledge on correct condom use, stigma in condom purchase and gendered power dynamics in condom use negotiation [10, 17]. Scholarly studies further point to greater control over condom use by males when compared to their female counterparts in most SSA countries. For instance, after investigating the experiences of South African men living with HIV/AIDS regarding condom use with their partners, Mfecane argued that entrenched gender power inequalities constitute the difficulties and constraints most women in SSA face with regards to condom use negotiation [18]. Women are largely expected to meet the sexual demands of their partners with little-to-no-decision-making power concerning their own sexual and reproductive health. Upon evaluation of how gendered power dynamics frame women’s and couples’ negotiations of contraceptive use in western Kenya, it was discovered that perceived and/or expressed male resistance was a major challenge to most women [19]. They further indicated that women who avoided male reproductive decision-making authority through covert contraceptive use, had concerns about severe consequences (e.g., divorce, violence) should their partners find out [19].

In Burkina Faso, although there exists a high-rate of HIV prevalence (i.e., 0.7%) among adult women aged 15–49 in comparison to the men (0.4%) in the same age category, the rate of condom use for high sexual risk reduction remains very low amongst women relative to men [20]. Recent studies have unveiled Burkina Faso’s women lack of autonomy regarding contraceptive use given the patriarchal and gender-unequal nature of society [21, 22]. For example, it was found that 47% of married women in the county could not refuse sexual intercourse with their husbands, and 62.9% did not have the ability to demand the use of condoms [23]. These unequal power dynamics have played out in Burkina Faso for many years, and women have struggled to receive equal treatment [24] or the overall empowerment needed to negotiate safer sex and condom use [23]. The implication is that women in polygamous and, or unfaithful relationships, and lack the needed empowerment to demand safe sex, stand the risk of contracting HIV and other STIs. Notwithstanding these gendered disparities in HIV prevalence and condom use for sexual risk reduction, comprehensive studies are yet to be conducted exploring the factors influencing condom use for HIV prevention among women in Burkina Faso. Our study fills this scholarly gap through the use of nationally representative data (i.e., Demographic and Health Survey) for cross-sectional analysis. Given the entrenched gendered power dynamics in most SSA countries, this study is also interested in understanding how women’s confidence to negotiate condom use may have an influence on condom usage in this context.

## 2.0 Theoretical framework

The Health Belief Model (HBM) was originally developed in the early 1950s by Hochbaum and colleagues [25] to understand the behaviour of people regarding the adoption of preventive measures against infections and diseases. The model theorized that health behaviours or the adoption of specific health behaviours are influenced by the desire to prevent illness and diseases [26]. HBM consists of four main dimensions, including: (a) perceived susceptibility to illness—the individual’s subjective susceptibility of contracting a disease; (b) perceived illness severity—an evaluation of both the medical/clinical consequences of the disease (e.g., pain, disability, death) and the possible social consequences (e.g., stigma and social rejection) that may result from contracting diseases like HIV/AIDS; (c) perceived benefits of treatment—the beliefs surrounding the effectiveness of health actions such as consistent condom use for reducing the risk of STIs; and (d) perceived barriers to treatment—a cost benefit analysis whereby the individual weighs the action’s effectiveness against possible negative consequences [26]. Cue to action is an added component of the HBM, which refers to the information required to trigger the process of engaging in healthy actions (e.g., knowledge about STIs and condom use for protection). Using HBM, this study, seeks to evaluate the factors associated with condom use by for protection against HIV infection among women in Burkina Faso. Based on our review of the literature and our theoretical construct in the HBM, we hypothesize that women with higher levels of social and socioeconomic empowerment and knowledge of HIV and other STIs are more likely to practice safe sex.

## 3.0 Study context

Burkina Faso is a landlocked West African nation that shares boundaries with Mali, Niger, Benin, Togo, Ghana, and Côte d’Ivoire. It has a total land area 274,200 km^2^. Majority of the country’s population resides in rural areas [27, 28]. Burkina Faso is a poor Sahelian nation with few resources—over 40% of its population are poor. According to the 2021–2022 UNDP Human Development Index (HDI) report, Burkina Faso ranks 184^th^ position out of 191 nations [29]. Healthcare access in Burkina Faso is a major challenge given its inadequate health infrastructure and lack of proximity to health facilities by majority of its population [30]. Existing inequities in maternal health services in Burkina Faso further jeopardizes the achievement of Universal Health Coverage in the country [31]. The weak economy and unstable nature of the country’s political landscape further increases the vulnerability of its citizens, particularly, women, to preventable disease and infections like HIV, gonorrhea, chlamydia, and syphilis among others. Consequently, women in Burkina Faso carry a disproportionate burden of HIV with low levels of condom use for risk reduction [20].

## 4.0 Materials and methods

### 4.1 Data collection

This study utilized data from the women’s file of the 2021 Burkina Faso Demographic and Health Survey (DHS), the fifth Demographic and Health Survey of Burkina Faso (EDSBF-V) carried out by the National Institute of Statistics and Demography (INSD) in collaboration with the National Institute of Public Health (INSP) from July 30 to November 30, 2021. The survey protocols, including all data collection tools, measurement procedures, and biological tests, were examined and approved by the National Ethics Committee of Burkina Faso and the Ethics Committee (Institutional Review Board) of International Coaching Federation (ICF). The 2019 Burkina Faso mapping base prepared by INSD for the General population and housing census in 2019 (RGPH 2019), constituted the sampling frame for drawing the study sample. The mapping base is a complete list of enumeration areas (EAs) which were created for the purposes of the census. It contains 23,663 ZDs and information on their identifier, their place of residence (urban or rural) and their household size counted, and their population recorded. Each ZD has a map delimiting its position and limits with documents of description.

The sampling frame was designed to ensure adequate representativeness of the main indicators (apart from adult mortality and maternal mortality) It was a stratified, two-stage area survey. The urban part and the rural part of each region correspond to a sampling stratum. In total, 26 sampling strata were created. The first stage of sampling included 195 clusters located in urban areas and 405 in rural areas selected to be mapped and investigated. At the end of the first-degree draw, a mapping and household enumeration operation was carried out. This operation, which targeted all 600 clusters, was finally carried out in 572 clusters, which made it possible to establish a situation plan and a sketch. The listing of households in the sample clusters was carried out using tablets, which also made it possible to record the GPS coordinates of the clusters and concessions. At the second level, 32 households per cluster in the Sahel region and 26 households in all other regions were systematically sampled. In the selected households, all women aged 15–49 years living there, or having spent the night before the interview, were eligible to take part in the survey. The 2019 Burkina Faso mapping base prepared by INSD for the General population and housing census in 2019 (RGPH 2019), constituted the sampling frame for drawing the study sample. The mapping base is a complete list of enumeration areas (EAs) which were created for the purposes of the census. It contains 23,663 ZDs and information on their identifier, their place of residence (urban or rural) and their household size counted, and their population recorded. Each ZD has a map delimiting its position and limits with documents of description.

The sampling frame was designed to ensure adequate representativeness of the main indicators (apart from adult mortality and maternal mortality) It was a stratified, two-stage area survey. The urban part and the rural part of each region correspond to a sampling stratum. In total, 26 sampling strata were created. The first stage of sampling included 195 clusters located in urban areas and 405 in rural areas selected to be mapped and investigated. At the end of the first-degree draw, a mapping and household enumeration operation was carried out. This operation, which targeted all 600 clusters, was finally carried out in 572 clusters, which made it possible to establish a situation plan and a sketch. The listing of households in the sample clusters was the carried out using tablets which also made it possible to record the GPS coordinates of the clusters and concessions. At the second level, 32 households per cluster in the Sahel region and 26 households in all other regions were systematically sampled. In the selected households, all women aged 15–49 years living there, or having spent the night before the interview, were eligible to take part in the survey. Visit https://dhsprogram.com/methodology/survey/survey-display-562.cfm for additional information on data collection. The underlying data for this study can be directly accessed at https://doi.org/10.5683/SP3/NADI8G

### 4.2 Ethical statement

Prior to the collection of data from women aged 15–49 years old, the National Institute of Statistics and Demography (INSD) of Burkina Faso received their informed consent, verbally. Where blood samples were taken from children aged 6 to 59 months, parents or guardians have given their consent to this effect. Also, authors have been granted permission to access and use the DHS data for research purposes.

### 4.3 Measures

#### Dependent variable

The outcome variable for this study was derived from a question that enquired whether participants reduce the risk of getting HIV through consistent condom use for sexual intercourse (0 = No, 1 = Yes).

#### Independent variable

The focal independent variable was derived from a question that enquired whether participants had the ability to negotiate condom use with their partners for sexual intercourse. All respondents had the option to answer “yes”, “no” and “it depends”. Based on these responses, “no” and “it depends” were regrouped as *no confidence to negotiate condom use* while “yes” was retained as *confidence to negotiate condom use* (0 = No confidence to negotiate, 1 = Confidence to negotiate). This regrouping was informed by earlier studies on condom use negotiation among women [32]. To account for possible confounders, other predictor variables were included and categorized under *Sociodemographic variables*: Level of education (0 = No education, 1 = Primary, 2 = Secondary, 3 = Higher), wealth (1 = Poorest, 2 = Poorer, 3 = Middle, 4 = Richer, 5 = Richest), ages (ranging from 15–49 years), religion (0 = Islam, 1 = Catholic, 2 = Protestant, 3 = Traditional/animist, 4 = No religion/Other); employment status (0 = Not employed, 1 = Employed). *Knowledge and risk variables*: Ever heard of sexually transmitted infections (0 = No, 1 = Yes); a healthy-looking person can have HIV (0 = No, 1 = Yes); ever been tested for HIV (0 = No, 1 = Yes); in a multiple partnership (0 = No, 1 = Yes). *Geographical variables*: Region of residence (1 = Boucle du Mouhoun, 2 = Cascades, 3 = Centre, 4 = Centre-Est, 5 = Centre-Nord, 6 = Centre-Ouest, 7 = Centre-Sud, 8 = Est, 9 = Hauts-Bassins, 10 = Nord, 11 = Plateau-Central, 12 = Sahel, 13 = Sud-Ouest), and type of place of residence (1 = Urban, 2 = Rural).

### 4.4 Analytical approach

We used descriptive statistics to provide the distribution of all the study variables. Furthermore, logistic regression models were employed to examine the association between the predictor variables and the outcome variable (i.e., condom use for HIV prevention). Given that the outcome variable is binary, a logistic regression was deemed more appropriate. Thus, a binary logistic regression was conducted for binary analysis, and multiple logistic regression for multivariate analysis. The results of these regression models are presented as odds ratios (OR). Significant odds ratios above one (OR > 1) indicate the likelihood of condom use, while odds ratios below one (OR < 1) indicate no likelihood of condom use. All statistical data analyses were conducted using Stata version 18.

## 5.0 Results

### 5.1 Sample characteristics

Table 1 presents the results for sample characteristics. From the results, majority of the women had no formal education (58.07%), were from Islamic religious backgrounds (62.80%), and resided in rural communities (64.74%). With regards to socioeconomic status, majority (59.88%) were employed with a significant proportion (54.26%) belonging to wealthy households (i.e., richer, and richest households). Although majority (77.46%) indicated being in multiple sexual relations, the had never heard of STIs (61.80%), and lack confidence to negotiate condom use (61.94%) with their partners. Also, a greater percentage of them (52.63%) never tested for HIV but knew that HIV can be asymptomatic (66.50%).

**Table 1:** Descriptive statistics table.

VARIABLES	Frequency (%)
**Respondent can ask partner to use a condom**	
No	10937(61.94)
Yes	6632(38.06)
**Education**	
No education	10254(58.07)
primary	2515(14.24)
secondary	4548(25.75)
higher	342(1.94)
**Wealth**	
Poorest	2869(16.25)
Poorer	3185(18.04)
Middle	3613(20.46)
Richer	3816(21.61)
Richest	4176(23.65)
**Age**	Mean (28.7877), SD (9.656254), Min = 15, Max = 49)
**Religion**	
Islam	11090(62.80)
Catholic	4183(23.69)
Protestant	1441(8.16)
Traditional/Animist	766(4.34)
No religion/Other	179(1.01)
**Currently employed**	
No	7084(40.12)
Yes	10575(59.88)
**In multiple Sexual relations**	
No	398(22.54)
Yes	13678(77.46)
**Ever heard of a sexually transmitted**	
No	10736(61.80)
Yes	6923(38.20)
**A healthy-looking person can have HIV**	
No	4139(23.44)
Yes	11743(66.50)
Don’t know	1777(10.06)
**Ever been tested for HIV**	
No	9293(52.63)
Yes	8366(47.37)
**Region of residence**	
Boucle du Mouhoun	1490(8.44)
Cascades	925(5.24)
Centre	2102(11.90)
Centre Est	1599(9.05)
Centre Nord	1167(6.61)
Centre Ouest	1605(9.09)
Centre Sud	1137(6.44)
Est	948(5.37)
Hauts-Bassins	1917(10.86)
Nord	1375(7.79)
Plateau Central	1457(8.25)
Sahel	618(3.50)
Sud-Ouest	1,319 (7.47)
**Type of place of residence**	
Urban	6227(35.26)
Rural	11432(64.74)

SD = Standard Deviation, Min = Minimum, Max = Maximum

### 5.2 Bivariate results for HIV risk reduction

Bivariate analyses are presented in Table 2. Women with confidence to negotiate condom use (OR = 2.50, P<0.001, 95%CI: 2.11, 2.96) were significantly more likely to use condom for HIV prevention than those who lack confidence to negotiate condom use. Also, compared to women without formal education, those who had education [primary education (OR = 1.32, P<0.001, 95%CI: 1.150, 1.52); secondary education (OR = 2.70, P<0.001, 95%CI: 2.40, 3.05); Tertiary education (OR = 8.13, P<0.001, 95%CI: 4.10, 16.12)] were significantly associated with higher likelihood of condom use for HIV prevention. Women from poorer households (OR = 1.23, P<0.05, 95%CI: 1.03, 1.46), middle-income households (OR = 1.70, P<0.001, 95%CI: 1.44, 2.02), richer households (OR = 2.52, P<0.001, 95%CI: 2.12, 2.99), and richest households (OR = 1.11, P<0.001, 95%CI: 1.09, 1.13) significantly had higher odds of use condom for HIV prevention compared to those from the poorest households. Age (OR = 1.11, P<0.001, 95%CI: 1.09, 1.13) was significant and positively associated with condom use. Furthermore, being in multiple sexual relationships (OR = 1.58, P<0.001, 95%CI: 1.42, 1.75), awareness about STIs (OR = 3.56, P<0.001, 95%CI: 3.14, 4.04), ever been tested for HIV (OR = 2.49, P<0.001, 95%CI: 3.14, 4.04), and knowing that HIV can be asymptomatic (OR = 4.063, P<0.001, 95%CI:), were all significantly associated with higher odds of condom use in the study context. However, women who were uncertain/don’t know whether a healthy-looking person can have HIV (OR = 0.29, P<0.001, 95%CI: 3.58, 4.59), were significantly less likely to practice condom use for HIV risk reduction. Women from rural areas (OR = 0.57, P<0.001, 95%CI: 0.51, 0.64) were significantly less likely to use condom for HIV prevention compared to women from urban areas. More so, women from Centre Est (OR = 1.402, P<0.05, 95%CI: 1.04, 1.88) were significantly more likely to use condom for HIV prevention relative to those form Cascades (OR = 0.59, P<0.001, 95%CI: 0.44, 0.80), Centre Nord (OR = 0.53, P<0.001, 95%CI: 0.40, 0.71), Centre Ouest (OR = 0.51, P<0.001, 95%CI: 0.39, 0.66) Est (OR = 0.46, P<0.001, 95%CI: 0.34, 0.61), Hauts-Bassins (OR = 0.47, P<0.001, 95%CI: 0.36, 0.60), Nord (OR = 0.58, P<0.001, 95%CI: 0.44, 0.78), Plateau Central (OR = 0.61, P<0.001, 95%CI: 0.46, 0.80), and Sud-Ouest (OR = 0.37, P<0.001, 95%CI: 0.28, 0.48).

**Table 2:** Logistic regression models for HIV risk reduction.

LOGISTIC REGRESSION MODELS				
VARIABLE	Bivariate		Multivariate	
	OR(SE)	CI	OR(SE)	CI
**Respondent can ask partner to use a condom** (Ref: No)				
Yes	2.50(0.21) ***	2.11 2.96	1.57(0.15) ***	1.29 1.91
**Education** (Ref: no education)				
primary	1.32(0.09) ***	1.150 1.52	1.21(0.14)	0.95 1.54
secondary	2.70(0.16) ***	2.40 3.05	1.38(0.17) *	1.07 1.77
higher	8.13(02.83) ***	4.10 16.12	3.12(3.28)	0.39 24.54
**Wealth** (Ref: poorest)				
poorer	1.23(0.10) *	1.03 1.46	1.12(0.15)	0.85 1.47
middle	1.70(0.14) ***	1.44 2.02	1.56(0.22) **	1.17 2.08
richer	2.52(0.22) ***	2.12 2.99	1.94(0.31) ***	1.42 2.67
richest	2.49(0.21) ***	2.11 2.94	1.64(0.34) *	1.08 2.47
**Age**	1.11(0.010) ***	1.09 1.13	0.99(0.02)	0.95 1.03
R**eligion** (Ref: Islam)				
Catholic	1.12(0.07)	0.99 1.27	0.99(0.12)	0.77 1.28
Protestant	1.05(0.09)	0.87 1.26	1.02(0.19)	0.69 1.48
Traditional/animist	0.44(0.06) ***	0.33 0.57	0.65(0.15)	0.40 1.05
No religion/Other	0.22(0.05) ***	0.13 0.35	0.26(0.10) ***	0.12 0.56
**Respondent currently employed** (Ref: No)				
Yes	1.02(0.05)	0.92 1.13	1.23(0.11) *	1.02 1.49
**In multiple Sexual relations (**Ref: No)				
Yes	1.58(0.08) ***	1.42 1.75	0.88(0.17)	0.60 1.30
**Ever heard of a sexually transmitted infection** (Ref: No)				
Yes	3.56(0.22) ***	3.14 4.04	1.58(0.17) ***	1.26 1.97
**A healthy-looking person can have HIV** (Ref: No)				
Yes	4.06(0.25) ***	3.58 4.59	2.64(0.27) ***	2.15 3.24
Don’t know	0.29(0.02) ***	0.24 0.35	0.34(0.05) ***	0.25 0.46
**Ever been tested for HIV** (Ref: No)				
Yes	2.49(0.15) ***	3.14 4.04	1.85(0.18) ***	1.52 2.24
**Region of residence** (Ref: Boucle du Mouhoun)				
Cascades	0.59(0.09) ***	0.44 0.80	0.27(0.07) ***	0.16 0.46
Centre	0.86(0.11)	0.66 1.11	0.45(0.12) **	0.27 0.76
Centre Est	1.40(0.21) *	1.04 1.88	0.64(0.16)	0.39 1.06
Centre Nord	0.53(0.07) ***	0.40 0.71	0.57(0.14) *	0.35 0.93
Centre Ouest	0.51(0.06) ***	0.39 0.66	0.50(0.12) **	0.31 0.81
Centre Sud	1.24(0.20)	0.90 1.72	0.80(0.23)	0.45 1.43
Est	0.46(0.06) ***	0.34 0.61	0.80(0.19)	0.49 1.29
Hauts-Bassins	0.47(0.06) ***	0.36 0.60	0.30(0.07) ***	0.19 0.48
Nord	0.58(0.08) ***	0.44 0.78	0.65(0.15)	0.40 1.05
Plateau Central	0.61(0.08) ***	0.46 0.80	0.75(0.19)	0.46 1.25
Sahel	0.99(0.18)	0.69 1.42	1.07(0.30)	0.61 1.86
Sud-Ouest	0.37(0.05) ***	0.28 0.48	0.30(0.07)	0.18 0.49
Not dejure resident	0.63(0.14) *	0.40 0.98	0.48(0.18)	0.22 1.03
**Type of place of residence** (Ref: Urban)				
Rural	0.575(0.032) ***	0.51 0.64	0.931(0.128)	0.71 1.22

*P<0.05

**P<0.01

***P<0.001

Odd Ratio (OR), Standard Error (SE), Confidence Interval (CI)

### 5.3 Multivariate results for HIV risk reduction

The results for multivariate analysis are also presented in Table 2. The results indicates that women with confidence to negotiate condom use with their partners (OR = 1.57, P<0.001, 95%CI: 1.29, 1.91), those who had secondary education (OR = 1.38, P<0.05, 95%CI: 1.07, 1.77), those from wealthy households [(middle-income (OR = 1.56, P<0.01, 95%CI: 1.17, 2.08), richer (OR = 1.94, P<0.001, 95%CI: 1.42, 2.67), richest (OR = 1.64, P<0.05, 95%CI: 1.08, 2.47)], employed women (OR = 1.23, P<0.05, 95%CI: 1.02, 1.49), women with knowledge of sexually transmitted infections (OR = 1.58, P<0.001, 95%CI: 1.26, 1.97), those ever been tested for HIV (OR = 1.85, P<0.001, 95%CI: 1.52, 2.24), and those who knew that HIV can be asymptomatic (OR = 2.64, P<0.001, 95%CI:) were all significantly more likely to practice consistent condom use for HIV prevention. However, women who were uncertain as to whether HIV can be asymptomatic (OR = 0.34, P<0.001, 95%CI: 2.15, 3.24), were significantly less likely to practice consistent condom use for HIV prevention. With regards to religious affiliation, those without religion (OR = 0.26, P<0.001, 95%CI:) were significantly less likely to practice use condom for HIV prevention. Moreover, regional disparities were observed. For instance, compared to Boucle du Mouhoun region, women from Cascades (OR = 0.27, P<0.001, 95%CI: 0.16, 0.46), Centre (OR = 0.45, P<0.01, 95%CI: 0.2, 0.76), Centre Nord (OR = 0.57, P<0.05, 95%CI: 0.35, 0.93), Centre Ouest (OR = 0.50, P<0.01, 95%CI: 0.3, 0.81), and Hauts-Bassins (OR = 0.30, P<0.001, 95%CI: 0.19, 0.48) were all less likely to use condom for HIV prevention.

## 6.0 Discussion

In this study, we explored the factors associated with condom use for HIV risk reduction amongst women of Burkina Faso. The study revealed that confidence to negotiate condom use with partners, having secondary education, coming from the richest households, being employed, having knowledge of sexually transmitted infections, ever been tested for HIV, and knowing that a healthy-looking person can have HIV, were all factors that significantly influence the ability of women to practice safe sex for HIV risk reduction.

The findings from our study revealing that women in Burkina Faso who lack confidence to negotiate the use of condoms with their male partners are less likely to practice consistent condom use for HIV risk reduction, aligns with previous findings in Tanzania [32]. In our study context, several factors, including gender norms and power dynamics, as well as the fear of partner rejection or partner violence may account for women’s lack of confidence to dialogue condom use with their partners during sexual intercourse. In the patriarchal society of Burkina Faso, just like other Sub-Saharan African (SSA) countries, there exist deeply ingrained gender norms and power imbalances [33]. Traditional gender roles typically place men in positions of authority and decision-making power [34, 35], leaving women with less control over their sexual and reproductive health decisions [36]. Moreover, the fear of rejection or partner violence tends to discourage most women from negotiating condom use. For instance, suggesting condom use to one’s partner may be interpreted as distrust, an accusation of promiscuity or infidelity and can lead to negative reactions from the male partner. This finding underscores the urgent need for interventions that aim to increase women’s confidence to ask for condom use during sexual intercourse by working to reduce the power imbalance within households that prioritizes men’s sexual reproduction decisions over that of women. We further observed that, educational attainment and religious affiliation were significantly associated with condom use for HIV risk reduction, a finding that aligns with earlier studies in the SSA context [37]. These findings can be explained by the fact that within SSA, there exist limited access to comprehensive sexual education and information on HIV/AIDS and other STIs. This works to hinder women’s ability to make informed decisions about their sexual health including condom use for STIs prevention. In the few contexts where such services are provided through community health education events, women with formal education are more likely than their uneducated counterparts to understand and practice the information received [38, 39]. Aside the imperative role that education plays on societal transformation, it also enhances women’s self-esteem, boosts their confidence, and ultimately build their freedom of expression on their sexual health [40]. Educated women are also more inclined to utilize media and other online health information sources as a guide in making sexual health decisions. This finding thus highlights the need to promote female education in Burkina Faso and similar contexts across SSA. On the grounds of religious affiliations, contrary to the findings of previous research [41, 42], our study shows that women without religious affiliation were less likely to adopt condom use for HIV prevention. Although Islam (62.8%) and the Catholic church (23.69%) were the most observed religions by participants in the study context, both religious faiths have historically refuted family planning and contraceptive methods [43, 44] on religious grounds. Thus, this finding is suggestive of change or advancement in religious teachings regarding sex education and reproductive health. Religious bodies are increasingly becoming a hub of sexual health communication where Some of them invite health professionals to talk to their congregants on sexual health and STIs. This may, over time, work to increase their awareness of HIV prevention relative to those without religious affiliation who do not have such opportunities. More coordinated efforts are needed to promote the teaching and sexual reproductive health by religious institutions. Moreover, socioeconomic factors, including household wealth and the employment status of women, were significantly associated with their condom use for HIV risk reduction. Prior studies indicate that women who depend on their partners to meet their financial needs tend to be hesitant towards condom use for fear of jeopardizing their economic security [37]. In this study, employed women may have been financially independent which possibly empowered them to practice condom use for HIV risk reduction. Similarly, women from wealthy households may have had better social support and access to financial resources and therefore, are likely to be financially independent. Financial autonomy has proven to have a positive impact on women’s decision-making power elsewhere [45]. Therefore, there is a need for the identification and empowerment of women from lower financial backgrounds to ensure they feel confident enough to negotiate condom use with their sexual partners. Cue to action, women who had ever been tested for HIV, those who knew that HIV can be asymptomatic, as well as those with awareness of STIs were significantly more likely to practice condom use for HIV risk reduction in the study context. While some people may feel invisible to HIV, especially if they had unprotected sexual intercourse in the past, women who have tested for HIV, particularly those who tested negative, would be more inclined to practice condom use for protection against infection as pre and post counselling sessions are mandatory in some countries. This counselling works to heighten their perceived risk of HIV infection. Likewise, women, with the understanding that HIV can be asymptomatic, would be more cautious and most likely resort to consistent condom use for protection against sexual transmission of the virus. This reinforces the need to intensify adult sexual health education, especially among uneducated women. Generally, sex education plays a crucial role in creating awareness about STIs, their symptoms and modes of transmission. However, throughout Sub-Saharan Africa, religious leaders and other stakeholders argue against the teaching of teenagers about sex and reproductive health at early ages despite growing consensus on the importance of equipping youth with the information they need to make smart and safe sexual choices [46, 47]. In Burkina Faso, women typically obtain their sexual health education from the mass media, and available evidence indicates that less than 20% of adolescents received their sexual health education in schools [47]. As a next step, it is important to provide or reinforce school-based educational opportunities that build the sexual health knowledge of girls in Burkina Faso at a young age. Also, regional differences were observed in consistent condom use for HIV risk reduction. Women who lived in Cascades, Centre, Centre Nord, Center Ouest and Hauts-Bassins were all significantly less likely to practice condom use for HIV prevention compared to women from the Boucle du Mouhoun region. Although Burkina Faso has undertaken a massive reform of its health system in the year 1992 through decentralization of its health care [48], health resources remain inequitably distributed across the regions as the urban regions were privileged at the expense of the rural and more remoted regions, especially regarding health personnel and health infrastructures [49]. For instance, during this health reform, the density of health personnel remained far below the World Health Organization (WHO) norms set at 23 per 10 000 capita. Even in 2014, the Centre Sud region which presented the highest health personnel ratio was still far below the WHO standard [49]. Nonetheless, health professionals are the most prominent source of reproductive health information, especially for uneducated women in remote areas. There is, therefore, the need for a government-led intervention to bridge the regional health disparities for equitable health care and access in Burkina Faso. There is a noteworthy limitation to this study. We observe that, the cross-sectional nature of the study limits the interpretation of the results to statistical associations. That notwithstanding, this study has made significant contributions to existing literature on preventive healthcare, particularly, HIV risk reduction. The results of the study also present some policy implications. First, given that women with secondary education, those from richer households, and those with knowledge of sexually transmitted infections (STIs) were more likely to practice condom use for HIV risk reduction, policy implications could involve the implementation of targeted educational programs. These programs should focus on enhancing awareness and education about STIs, HIV transmission, and the importance of condom use. Special attention could be directed towards women with lower educational attainment and those from poor economic backgrounds. Also, empowerment initiatives are crucial since women who had the confidence to negotiate condom use with their partners were more likely to practice condom use. The finding highlights the importance of empowerment in sexual health decision-making. Policies aimed at empowering women in relationships, enhancing their communication skills, and fostering self-confidence could contribute to increased condom use for HIV risk reduction. This may involve incorporating relationship and communication skills training into educational and sexual health programs. More so, the study indicates that women who had ever been tested for HIV were significantly more likely to practice condom use for HIV risk reduction. Therefore, policies should focus on improving the availability of, and accessibility to HIV testing services. This might involve promoting regular HIV testing, reducing barriers to testing, and providing information about the benefits of knowing one’s HIV status. Geographical considerations should also be considered to ensure equitable access to testing services across different regions.

To sum up, the policy implications of the study findings revolve around targeted education, empowerment initiatives, and improvements in the accessibility of HIV testing services. Implementing these policies can contribute to promoting safer sexual practices and reducing the risk of HIV transmission among women, particularly those in vulnerable or disadvantaged situations.

## 7.0 Conclusion

This study points to confidence to negotiate condom use, educational attainment, religious background, household wealth, employment status, multiple sexual relationships, HIV testing, knowledge of STIs, and geographical location as factors that influence the practice of safe sex for HIV risk reduction among women of reproductive age in Burkina Faso. Globally, efforts are being made by various organizations, both local and international, to promote gender equality, provide education and awareness campaigns, improve access to contraceptives, and offer support services for women to empower them to negotiate condom use and to make informed choices about their sexual health. It is important to recognize that addressing deeply entrenched cultural norms and complex societal issues takes time and concerted effort from multiple stakeholders.
